# Medical waste management within the Green Hospital approach: a systematic review of direct, contextual, and transferable evidence

**DOI:** 10.1093/intqhc/mzag103

**Published:** 2026-08-03

**Authors:** İbrahim H Kayral, Yankuba B Manga, Şeyma Boz, Peter Lachman, Usman Iqbal

**Affiliations:** Hospitals Quality Coordination, Hacettepe University, Ankara, Türkiye; Department of Health Management, Faculty of Health Sciences, İzmir Bakırçay University, İzmir, Türkiye; Master Program in Smart Healthcare Management, Master Program in Artificial Intelligence Applications and Management, and Bachelor Program in Smart Sustainable Development and Management, International College of Sustainability Innovations, National Taipei University, New Taipei City, Taiwan; Health Institutes of Türkiye (TÜSEB), İstanbul, Türkiye; Patient Safety Movement Foundation, Dublin, Ireland; Institute for Evidence-Based Healthcare, Faculty of Health Sciences & Medicine, Bond University, Gold Coast, QLD, Australia; Evidence-Based Practice Professorial Unit, Gold Coast Hospital & Health Service (GCHHS), Gold Coast, QLD, Australia; Centre for Data Analytics, Bond Business School, Bond University, Gold Coast, QLD, Australia; School of Population Health, Faculty of Medicine and Health, University of New South Wales, Sydney, NSW, Australia

**Keywords:** Medical waste, Green hospitals, Waste segregation, Digital health, Environmental management

## Abstract

**Background:**

Medical waste management is a central pillar of the Green Hospital approach, yet evidence on effective strategies and system-level barriers remains fragmented. This systematic review synthesizes direct medical-waste-management evidence together with healthcare-sustainability and transferable waste-system evidence relevant to the Green Hospital framework.

**Methods:**

We conducted a systematic review in accordance with PRISMA 2020. The review protocol was registered in PROSPERO (CRD420251168204). Six bibliographic databases and one grey-literature source were searched for original empirical, intervention, case-study, modelling, and optimization studies published online between 1 January 2020 and 27 August 2025 that examined medical waste management or provided clearly relevant healthcare-sustainability or transferable waste-system evidence. Two reviewers independently screened records, extracted data, and appraised methodological quality using design-appropriate criteria: the Mixed Methods Appraisal Tool for empirical studies and an adapted model-appraisal framework for modelling, optimization, framework-development, and technology-evaluation studies.

**Results:**

Of the 21 included studies, 15 provided direct medical-waste-management evidence, five provided healthcare-sustainability or planetary-health contextual evidence, and one provided transferable waste-system evidence. Behavioural compliance, education, or segregation was addressed in 11 studies (52%); workflow or operational optimization in four studies (19%); digital or decision-support systems in five studies (24%); environmental or economic assessment, modelling, circularity, or resource recovery in 10 studies (48%); and systemic or organizational barriers in seven studies (33%). Studies could contribute to more than one synthesis theme; therefore, these categories were not mutually exclusive.

**Conclusion:**

Sustainable medical waste management within the Green Hospital framework requires an integrated socio-technical strategy combining behavioural reinforcement, operational improvement, decision-support technologies, environmental and economic assessment, and enabling governance. These findings can be interpreted through a Human Factors and Ergonomics (HFE) lens, although formal HFE interventions were not consistently evaluated in the included studies.

## Introduction

The environmental impact of healthcare institutions has gained increasing attention within global sustainability frameworks during the past two decades. Hospitals represent one of the most resource-intensive service sectors, contributing significantly to environmental degradation through high energy consumption, intensive water use, and substantial waste generation. According to the World Health Organization [1], ∼15% of hospital waste is classified as hazardous, posing risks such as infection, cytotoxic exposure, and chemical toxicity. In this context, the “Green Hospital approach” has emerged as a fundamental paradigm supporting environmentally responsible healthcare delivery.

The Green Hospital model encompasses a broad set of practices, including energy efficiency, carbon emission reduction, sustainable procurement, resource conservation, and medical waste management as a critical component [2].

Post-2022 studies have highlighted increasing use of plastic, inadequate segregation practices, and carbon-intensive processes that hinder sustainability efforts [3, 4]. Evidence from Türkiye and other developing contexts has shown that gaps in staff training, behavioural barriers, and infrastructural limitations directly affect waste management performance.

Despite growing interest, previous reviews have either (i) focused on pre-COVID-19 evidence, (ii) examined individual components such as training or waste minimization in isolation, or (iii) failed to integrate findings within the broader Green Hospital and circular-economy frameworks. This review conceptualizes medical waste management within the Green Hospital framework as a multidimensional system comprising behavioural compliance, technological enablement, and organizational capacity.

## Materials and methods

### Protocol and registration

This systematic review was reported in accordance with the PRISMA 2020 statement. The review protocol was registered in PROSPERO (CRD420251168204).

### Information sources and search strategy

A comprehensive literature search was conducted across six bibliographic databases—MEDLINE (PubMed), Web of Science, Scopus, Embase, the Cochrane Library, and CINAHL (EBSCO), and one grey-literature source, OpenGrey, with the final search completed on 27 August 2025. Publication eligibility was determined according to the date on which an article first became available online, irrespective of its subsequent issue assignment. All records were exported to Endnote (v2025) for both automated and manual deduplication. The reference lists of included studies were screened to identify additional eligible articles. Search strategies incorporated controlled vocabulary (e.g. MeSH terms) and free-text keywords related to medical waste management, environmental sustainability, and the Green Hospital framework. Database-specific search strings and Boolean combinations are delineated in Supplementary File S1, while a structured overview of the PubMed search is presented in Supplementary Table S1.

### Eligibility criteria

Studies were eligible if they were original empirical, intervention, case-study, systems-modelling, or optimization investigations published online in English between 1 January 2020 and 27 August 2025 and met at least one of the following criteria: (i) directly examined medical or healthcare waste generation, ­segregation, collection, storage, transport, treatment, disposal, recycling, cost, or governance in healthcare settings; (ii) evaluated sustainability-oriented behavioural, educational, technological, circular-economy, supply-chain, or environmental interventions with clear relevance to healthcare operations; or (iii) provided transferable waste-system evidence applicable to sustainable healthcare waste management. Eligible outcomes included environmental, behavioural, organizational, operational, healthcare-related, economic, regulatory, or resource-efficiency outcomes. Reviews, commentaries, editorials, protocols, conference abstracts without sufficient data, and studies without a defensible relationship to medical-waste management or sustainable healthcare were excluded. Because the included evidence differed in its proximity to medical-waste management, studies were classified as direct medical-waste-management evidence, healthcare-sustainability contextual evidence, or transferable waste-system evidence.

### Study selection

Two reviewers independently screened titles and abstracts utilizing predefined eligibility criteria. The full texts of potentially relevant studies were subsequently assessed independently. Full texts were sought through institutional library subscriptions, publisher websites, academic search engines, and other available document-retrieval channels. Reports that remained inaccessible after these attempts were classified as not retrieved. The high number of unretrieved reports partly reflected the deliberately broad, high-sensitivity search, particularly the large yield from the Cochrane Library. Reports were classified as not retrieved only when no accessible full text could be located through the available retrieval channels; the review log did not further subdivide the reasons for non-retrieval. Discrepancies were resolved through discussion or adjudication by a third reviewer. The overall identification, screening, and inclusion process adhered to PRISMA 2020 guidelines and is illustrated in the accompanying PRISMA flow diagram. Full database-specific search strings are provided in Supplementary File S1.

### Data extraction

Data extraction was conducted independently by two reviewers utilizing a piloted data extraction form. Extracted information included study design, country or context, setting, sample size or analytical unit, intervention or focus area, key outcomes, principal findings, and evidence relevance. Studies were classified as direct medical-waste-management evidence, healthcare-sustainability contextual evidence, or transferable waste-system evidence. Outcomes were coded across behavioural, operational, digital or decision-support, environmental or economic, and systemic or organizational themes. Any discrepancies were resolved through consensus or consultation with a third reviewer.

### Quality assessment

The methodological quality of included studies was appraised independently by two reviewers using design-appropriate ­criteria. Empirical studies were assessed using the Mixed Methods Appraisal Tool (MMAT) [37], with the relevant criteria applied according to study design: randomized controlled, non-randomized, or quantitative descriptive. Modelling, optimization, framework-development, and technology-evaluation studies were assessed using an adapted model-appraisal framework informed by published good-practice guidance for decision-analytic models [38]. The framework covered clarity of objectives and scope, appropriateness of the model structure, transparency and suitability of data inputs, reporting of assumptions, validation, uncertainty or sensitivity analysis, and applicability of the findings. Each criterion was rated as ‘Yes’, ‘No’, or ‘Cannot tell’. No aggregate numerical score or percentage was calculated. Disagreements between reviewers were resolved through discussion or consultation with a third reviewer.

### Data synthesis

Owing to substantial heterogeneity in study designs, populations, interventions, analytical approaches, and outcome measures, meta-analysis was not appropriate. A structured narrative synthesis was therefore conducted. Studies were first classified according to evidence relevance as direct medical-waste-management evidence, healthcare-sustainability contextual evidence, or transferable waste-system evidence. Findings were then coded across five non-mutually exclusive synthesis themes: behavioural compliance, education, or segregation; workflow or operational optimization; digital, internet of things (IoT)-enabled, analytical, or decision-support systems; environmental or economic assessment, modelling, circularity, or resource recovery; and systemic, organizational, governance, infrastructure, or regulatory barriers. Studies could contribute to more than one theme. Contextual and transferable studies were used to inform the broader Green Hospital interpretation but were not treated as direct evidence of medical-waste-management effectiveness. Human Factors and Ergonomics (HFE) was not used as an eligibility criterion or a prespecified coding framework; it was applied as an interpretive socio-technical lens when discussing the synthesized findings.

### Ethical considerations

Ethical approval was not required for this review, as it did not involve human participants or primary data collection.

## Results

### Study selection

**Figure 1:**
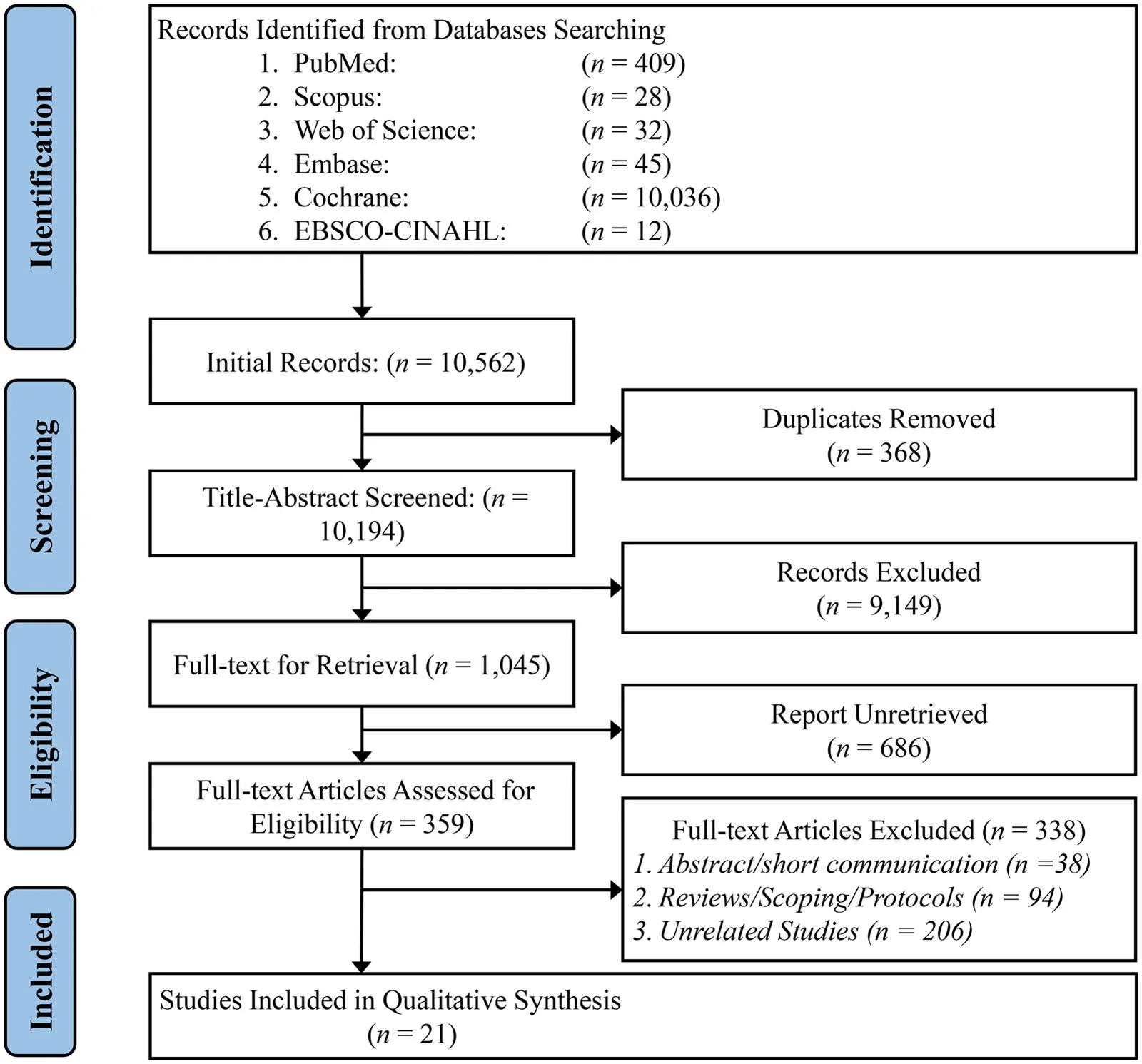
PRISMA flow diagram for the search and selection process of this study. OpenGrey was searched as a grey-literature source but yielded no records (*n* = 0); therefore, it is not shown as a separate source box in the flow diagram.

A total of 10 562 records were identified through database searching. After the removal of 368 duplicates, 10 194 records underwent title and abstract screening, of which 9149 were excluded. Reports were sought for retrieval for 1045 records; 686 reports could not be retrieved. The remaining 359 full-text reports were assessed for eligibility. Of these, 338 were excluded: unrelated studies (*n* = 206), reviews, scoping reviews, or protocols (*n* = 94), and abstracts or short communications (*n* = 38). Ultimately, 21 studies met the eligibility criteria and were included in the qualitative synthesis (Fig. 1).

### General characteristics of included studies

The evidence base comprised three individually randomized controlled trials, two cluster-randomized interventions, two non-randomized or quasi-experimental studies, five quantitative descriptive or cross-sectional studies, one combined observational waste-audit and cross-sectional study, and eight modelling, framework-development, optimization, or technology-evaluation studies. Fifteen studies provided direct evidence on medical or healthcare waste management, five provided healthcare-sustainability or planetary-health contextual evidence, and one provided transferable waste-system evidence. Settings included hospitals, operating rooms, microbiology laboratories, outpatient pharmacies, village clinics, primary-healthcare services, healthcare supply chains, public-hospital systems, municipal waste pilot sites, and community or planetary-health contexts.

Contextual studies reported sustainability-related behavioural, resilience, planetary-health, or lifestyle outcomes but did not directly measure medical-waste-management performance. Detailed study characteristics, evidence relevance, sample or analytical units, principal findings, and synthesis-theme mapping are presented in Table 1.

**Table 1:** Characteristics, key outcomes, and synthesis mapping of the studies included in the systematic review (*n* = 21).^a^

No.	Study	Country/Context	Design	Setting/Sample or analytical unit	Intervention/Focus	Key outcomes and principal findings	ER; Theme(s)
1	Adam *et al.*, 2025 [9]	Somalia	Nationwide cross-sectional survey	423 healthcare facilities	Segregation and disposal of infectious and sharps waste	More than 60% of facilities lacked adequate segregation systems. Incineration was most frequently used for infectious waste (33.57%), whereas pit burning was common for sharps waste (52.48%). Significant regional inequalities were identified (*P* < .001).	MWM; B, S
2	Addas *et al.*, 2024 [20]	Pakistan	IoT-based pilot evaluation	Ten pilot locations in Lahore; >200 million data points	IoT-enabled fill-level monitoring, cloud analytics, and dynamic route optimization	Route efficiency improved by 32%, fuel use and emissions decreased by 29%, waste-processing throughput increased by 33%, and vehicle-maintenance costs decreased by 18%. The findings represent transferable waste-system evidence rather than healthcare-specific outcomes.	TWS; D, O, E
3	Alfina *et al.*, 2025 [21]	Indonesia	Mixed-method modelling using expert input, BN, and DBM	Healthcare supply-chain context; expert sample size NR	Circular-economy integration and dynamic risk management in healthcare supply chains	Optimized sourcing and operations increased high-impact sustainability from 38.6% to 52.2%. Resilience increased by 16.7%, public-health impact by 17.3%, reliability by 12.5%, responsiveness and flexibility by 38.8%, and asset utilization by 15.3%.	HSC; D, E, S
4	Atta *et al.*, 2025 [22]	Egypt	RCT	130 rural primary-healthcare nurses randomized; 124 completed the study	Web-based climate-change education targeting awareness, environmental self-efficacy, activism, and pro-environmental behaviour	Climate-change knowledge, environmental self-efficacy, and pro-environmental behaviour improved significantly in the intervention group at both post-intervention assessments (all *P* < .001). No direct medical-waste outcome was measured.	HSC; B
5	Bektaş *et al.*, 2024 [23]	Türkiye	Cross-sectional survey	140 operating-room personnel and medical students at a university hospital	Environmental sustainability, recycling, anaesthetic practices, and medical-waste awareness	Although 76% reported attention to waste segregation and 89% believed environmental impacts could be reduced, inadequate knowledge, negative attitudes, limited formal education, and insufficient recycling services remained major barriers.	MWM; B, S
6	Bicket *et al.*, 2021 [24]	USA	Multi-arm RCT	499 individuals recruited from two Johns Hopkins outpatient pharmacies; 227 reported leftover medication at follow-up	Disposal kits and fact sheets for unused prescription opioids	Safe disposal did not differ significantly between no intervention (10%), disposal-kit provision (14%), and fact-sheet provision (11%). Passive provision alone was insufficient to change disposal behaviour.	MWM; B
7	Çetin *et al.*, 2025 [25]	Türkiye	Observational waste audit and cross-sectional survey	Forty-five-day audit in six university-hospital laboratory units; 304 healthcare professionals	Waste generation, classification accuracy, regulatory compliance, and staff training	Mean daily generation was 22.78 kg of medical waste, 11.67 kg of liquid waste, and 5.61 kg of sharps waste. Fifteen per cent of personnel misclassified hazardous waste, and training frequency was the strongest predictor of compliance (*R*² = 0.72, *P* < .01).	MWM; B, E
8	D’Alessandro *et al.*, 2025 [3]	Italy	Framework development and healthcare case study	One private healthcare clinic; participant sample NA	Integration of ERA and MFA within the Deming Cycle	The framework enabled simultaneous assessment of material flows and environmental and health risks. The case study identified benefits for waste tracking, hotspot detection, and risk prioritization, alongside data and implementation barriers.	MWM; D, O, E
9	Dixit and Dutta, 2024 [4]	India	Mixed-method analytical study using survey analysis, ANOVA, ISM, and fuzzy DEMATEL	Indian healthcare organizations and expert respondents; *n* NR	Critical success factors for circular-economy adoption in healthcare-waste management	Of 17 critical success factors, 12 were classified as causal and five as effect factors. Government responsibility and stakeholder participation had the greatest driving influence, whereas segregation and collection depended on other organizational and policy factors.	MWM; D, E, S
10	Erdem, 2022 [26]	Türkiye	Mathematical optimization and logistics modelling	Real-life benchmark instances representing a hazardous medical-waste collection network	Electric-vehicle routing, charging decisions, energy costs, and transport risk	The model supported cost-effective, low-emission medical-waste collection. Mixed use of charger types reduced total energy costs and transport risk compared with reliance on a single charger type.	MWM; O, E
11	Evliya Felek *et al.*, 2025 [27]	Türkiye	Quasi-experimental study	53 operating-room staff and associated waste streams at Ankara University Cebeci Hospital	Waste-segregation training with immediate and 2-month follow-up assessments	Recyclable waste increased from 1.30 to 1.80 kg per operation after training (*P* = .01). Medical waste decreased from 4.92 to 4.14 kg per operation, although the reduction was not significant (*P* = .09). Benefits weakened at 2 months.	MWM; B, O
12	Eweida *et al.*, 2025 [28]	Egypt	RCT	140 nursing students from rural communities	Video-based climate-change education addressing climate literacy and environmental self-efficacy	Climate-change perceptions, pro-environmental attitudes, and environmental self-efficacy improved, while cognitive impairment associated with climate anxiety decreased. No direct medical-waste outcome was measured.	HSC; B
13	Gao *et al.*, 2022 [29]	China	Cross-sectional survey with on-site inspection	236 village clinics in 21 counties across three provinces	Medical-waste generation, segregation, collection, storage, disposal, and PPE use	Mean waste generation was ∼0.65 kg per clinic per day or 0.17 kg per patient per day. Most clinics did not segregate waste correctly; major barriers included inadequate funding, equipment, storage areas, and designated personnel.	MWM; B, S
14	Hamed *et al.*, 2025 [30]	Egypt	Quasi-experimental pre–post study	75 nurses at El-Minia University Hospitals	Three-session biomedical-waste education programme	Knowledge, attitudes, and practices improved significantly following the intervention (all *P* < .001). The association between knowledge and practice strengthened after training (*r* = 0.62, *P* < .001).	MWM; B
15	Moloisi and Onwubu, 2024 [31]	South Africa	Cross-sectional survey	34 waste handlers at Dr George Mukhari Academic Hospital	Knowledge of isolation-waste categories, colour coding, PPE, safety precautions, and institutional policies	Most participants correctly identified waste types, sharps, colour codes, and PPE requirements. Awareness of internal policies remained incomplete, and knowledge differed significantly according to age and experience.	MWM; B, S
16	Rohr *et al.*, 2023 [32]	Senegal	Cluster-randomized intervention with ecological, health, and economic assessments	Sixteen villages: eight intervention and eight control communities	Removal and reuse of aquatic vegetation to improve water access, reduce schistosomiasis, and recover resources	Control communities had 1.46 times higher intestinal schistosomiasis infection rates. Reuse of vegetation as livestock feed or compost generated benefit-to-cost ratios of up to 4.0 and 8.8, respectively. The study provided broader planetary-health and circular-resource evidence.	HSC; E
17	Sarwar *et al.*, 2025 [33]	International/model-based	MCDM model with comparative and sensitivity analyses	Medical-waste treatment technologies and evaluation criteria; expert-panel size NR	f, g, h-fractional fuzzy CRITIC–TOPSIS framework for treatment-technology selection	The model produced stable rankings across comparative and sensitivity analyses. The highest-ranked treatment alternative remained preferred across scenarios, supporting the robustness of the framework under uncertainty.	MWM; D, E
18	Sepetis *et al.*, 2022 [17]	Greece	Regression and predictive cost modelling	121 observations from Greek public hospitals for 2018	Prediction of hazardous healthcare-waste quantities and management costs	Public hospitals generated ∼9500 tonnes of hazardous healthcare waste in 2018, projected to exceed 18 200 tonnes in 2025 and 18 800 tonnes in 2030. Waste quantity and cost were associated with hospital characteristics and service activity.	MWM; E
19	Sepetis *et al.*, 2025 [34]	Greece	Environmental-cost analysis using OLS regression	119 of 125 Greek public hospitals	Environmental costs associated with energy, water, and waste management	Environmental costs represented a meaningful component of operating expenditure and were particularly high in university and specialist hospitals. Bed capacity, specialized units, and natural-gas use significantly influenced environmental costs.	MWM; E
20	Tushar *et al.*, 2023 [35]	Bangladesh/emerging-economy context	Expert-based BWM, ISM, and MICMAC modelling	Eight experts from hospitals, diagnostic centres, municipal organizations, and academia	Identification and causal mapping of barriers to sustainable medical-waste management	Seventeen barriers were validated and the 12 highest-priority barriers were structurally modelled. Weak law enforcement and inadequate financial support were the most critical barriers; training, technology, and coordination limitations were strongly interdependent.	MWM; S
21	Yu *et al.*, 2025 [36]	China	Cluster RCT	383 community-dwelling adults from 20 communities; 190 intervention and 193 control participants	Socioecological, smart-device-based lifestyle intervention	At 6 months, healthy-lifestyle scores improved modestly and more intervention participants reduced at least one unhealthy behaviour (31.48% vs 19.64%, *P* = .016). Effects were not sustained at 12 months. No medical-waste outcome was measured.	HSC; B

ANOVA, analysis of variance; BN, Bayesian network; BWM, best–worst method; DBM, dynamic barrier management; ISM, interpretive structural modelling; MCDM, multi-criteria decision-making; MICMAC, cross-impact matrix multiplication applied to classification; NA, not applicable; NR, not reported; OLS, ordinary least squares; PPE, personal protective equipment; RCT, randomized controlled trial.

a *Evidence relevance codes*: MWM, direct medical-waste-management evidence; HSC, healthcare-sustainability or planetary-health contextual evidence; TWS, transferable waste-system evidence. *Synthesis theme codes*: B, behavioural compliance, education, or segregation; O, workflow or operational optimization; D, digital, IoT-enabled, AI-supported, or decision-support systems; E, environmental or economic assessment, modelling, circularity, or resource recovery; S, systemic, organizational, governance, infrastructure, or regulatory barriers.

### Behavioural compliance, education, and waste segregation

Eleven studies contributed to the behavioural compliance, education, or segregation theme [9, 22–25, 27–31, 36]. Eight studies directly examined waste-related knowledge, segregation, disposal, or educational interventions in healthcare settings [9, 23–25, 27, 29–31]. These studies identified inadequate knowledge, limited training, weak institutional reinforcement, and insufficient infrastructure as recurring determinants of poor segregation or disposal performance. Educational interventions improved knowledge and practices, although the effects were not always sustained; for example, operating-room improvements weakened by the 2-month follow-up [27], while passive provision of opioid-disposal information or kits did not significantly improve safe disposal [24]. Three additional studies provided contextual evidence that digital or video-based education could improve environmental knowledge, self-efficacy, or pro-environmental behaviour, but they did not measure medical-waste outcomes directly [22, 28, 36].

### Workflow and operational optimization

Four studies contributed to the workflow and operational-optimization theme [3, 20, 26, 27]. Waste-segregation training altered the distribution of medical and recyclable waste in an operating-room setting, although the effect weakened over time [27]. Logistics modelling demonstrated potential reductions in energy costs, emissions, and transport risk through electric-vehicle routing and mixed charging strategies [26]. The environmental risk assessment (ERA)–material flow analysis (MFA) framework supported waste tracking, hotspot identification, and risk prioritization in a private healthcare clinic [3]. A transferable IoT-based municipal-waste pilot improved route efficiency, fuel use, emissions, throughput, and maintenance costs, although it did not evaluate healthcare waste directly [20].

### Digital, analytical, and decision-support systems

Five studies contributed to the digital, analytical, or decision-support theme [3, 4, 20, 21, 33]. These studies included IoT-enabled fill-level monitoring and route optimization [20], integration of ERA with MFA [3], Bayesian-network and Dynamic Barrier Management modelling for healthcare supply-chain resilience [21], interpretive structural modelling and fuzzy-DEMATEL (decision-making trial and evaluation laboratory) analysis of circular-economy adoption factors [4], and fractional fuzzy CRITIC (criteria importance through intercriteria correlation)–TOPSIS (technique for order preference by similarity to ideal solution) modelling for medical-waste treatment-technology selection [33]. Reported benefits included improved route efficiency, stronger risk prioritization, enhanced sustainability and resilience indicators, identification of high-driving organizational factors, and stable technology rankings. However, only a subset of this evidence was derived from healthcare-waste operations, and the included studies did not establish the effectiveness of AI-enabled segregation systems in routine hospital practice.

### Environmental and economic impact modelling

Ten studies contributed to the environmental or economic assessment, modelling, circularity, or resource-recovery theme [3, 4, 17, 20, 21, 25, 26, 32–34]. These studies identified hospital size, service intensity, and presence of specialized units as key drivers of waste management costs. Integrated environmental assessments reported the environmental burden associated with healthcare waste streams, while forecasting and logistics optimization models demonstrated potential reductions in emissions and operational costs under sustainability-oriented scenarios.

### Systemic barriers and organizational constraints

Seven studies contributed to the systemic and organizational-barriers theme [4, 9, 21, 23, 29, 31, 35]. Commonly reported challenges included weak regulatory enforcement, inadequate ­infrastructure, limited access to non-incineration technologies, insufficient funding, and gaps in continuous staff training.

### Methodological quality assessment

Study-level methodological appraisal is presented in Table 2. Among the randomized studies, randomization procedures and baseline comparability were generally adequate; however, completeness of follow-up and adherence were less consistent. The Bicket trial had substantial non-response at follow-up, although its primary analysis was prospectively restricted to participants who had discontinued opioid therapy and retained leftover medication. The Yu cluster trial reported increasing attrition and variable adherence over longer follow-up. The non-randomized intervention studies used appropriate outcome measures and reported complete post-intervention data, but neither adequately controlled for potential confounding. Among quantitative descriptive studies, multistage or near-population sampling strengthened the Adam, Gao, and Sepetis studies, whereas single-centre voluntary surveys had greater concerns regarding representativeness, non-response, or measurement validity. Modelling and optimization studies generally reported clear objectives and appropriate analytical structures; however, formal uncertainty or sensitivity analyses and external validation were inconsistently reported. No aggregate quality score was calculated.

**Table 2:** Study-level methodological appraisal using design-appropriate criteria.

Panel A. MMAT appraisal of empirical studies^a^ ^,^ ^b^							
Study	Design category	C1	C2	C3	C4	C5	Main methodological concern
Atta *et al.*, 2025 [22]	RCT	Y	Y	Y	Y	Y	Short follow-up and reliance on self-reported outcomes
Bicket *et al.*, 2021 [24]	RCT	Y	Y	N	Y	Y	Substantial non-response at follow-up; the primary analysis was appropriately restricted to participants with leftover medication
Eweida *et al.*, 2025 [28]	RCT	Y	Y	Y	CT	CT	Outcome-assessor blinding and intervention adherence not fully reported
Rohr *et al.*, 2023 [32]	Cluster-randomized intervention	Y	Y	Y	CT	Y	Blinding of outcome assessment not clearly reported
Yu *et al.*, 2025 [36]	Cluster RCT	Y	Y	CT	Y	CT	Attrition increased at 12 months and adherence varied across intervention components
Evliya Felek *et al.*, 2025 [27]	Non-randomized intervention	CT	Y	Y	N	Y	Single-centre design and important procedural confounders were not controlled
Hamed *et al.*, 2025 [30]	Non-randomized intervention	CT	Y	Y	N	Y	No control group and limited control of confounding
Adam *et al.*, 2025 [9]	Quantitative descriptive	Y	Y	Y	CT	Y	Non-response and facility-level missingness were not fully described
Bektaş *et al.*, 2024 [23]	Quantitative descriptive	CT	N	CT	CT	Y	Single-centre sample, unclear sampling frame, and possible non-response bias
Çetin *et al.*, 2025,[25]	Quantitative descriptive	CT	N	CT	CT	Y	Voluntary single-centre sample and partly self-reported questionnaire data
Gao *et al.*, 2022 [29]	Quantitative descriptive	Y	Y	Y	Y	Y	Findings remain limited to selected prefectures despite strong multistage sampling
Moloisi and Onwubu, 2024 [31]	Quantitative descriptive	Y	Y	CT	Y	Y	Small single-hospital sample and limited reporting of questionnaire validation
Sepetis *et al.*, 2025 [34]	Quantitative descriptive	Y	Y	Y	Y	Y	Limited by the available administrative data and the 2018–19 observation period

Panel B. Appraisal of modelling, optimization, framework-development, and technology-evaluation studies^c^ ^,^ ^d^								
Study	M1	M2	M3	M4	M5	M6	M7	Main methodological concern
Addas *et al.*, 2024 [20]	Y	Y	Y	CT	Y	N	Y	Large pilot dataset but no formal uncertainty or sensitivity analysis
D’Alessandro *et al.*, 2025 [3]	Y	Y	CT	Y	CT	N	Y	Illustrative single-clinic application; several inputs were literature-derived and sensitivity analysis was not performed
Alfina *et al.*, 2025 [21]	Y	Y	Y	Y	Y	Y	Y	Limited number of case contexts and reliance on expert input
Dixit and Dutta, 2024 [4]	Y	Y	Y	Y	Y	N	Y	No formal sensitivity analysis of the resulting causal rankings
Erdem, 2022 [26]	Y	Y	Y	Y	Y	Y	Y	Benchmark instances partly depended on assumptions and expert-derived parameters
Sarwar *et al.*, 2025 [33]	Y	Y	CT	Y	Y	Y	Y	Limited expert base may affect input weights and external generalizability
Sepetis *et al.*, 2022 [17]	Y	Y	Y	Y	CT	N	Y	Strong internal predictive performance but no external validation or uncertainty analysis
Tushar *et al.*, 2023 [35]	Y	Y	Y	Y	CT	N	Y	Small expert panel, subjective input, and no comparative sensitivity analysis

CT, cannot tell from the published report; N, no; RCT, randomized controlled trial; Y, yes.

a MMAT criteria.

b *Randomized studies*: C1, randomization appropriately performed; C2, groups comparable at baseline; C3, complete outcome data; C4, outcome assessors blinded; C5, participants adhered to the assigned intervention. *Non-randomized studies*: C1, participants representative of the target population; C2, measurements appropriate; C3, complete outcome data; C4, confounders accounted for; C5, intervention administered as intended. *Quantitative descriptive studies*: C1, sampling strategy relevant; C2, sample representative of the target population; C3, measurements appropriate; C4, risk of non-response bias low; C5, statistical analysis appropriate.

c *Model-appraisal criteria*: M1, objectives and scope clearly defined; M2, model structure and analytical approach appropriate; M3, data inputs transparent and suitable; M4, assumptions explicitly reported; M5, internal, external, comparative, or expert validation undertaken; M6, uncertainty or sensitivity analysis performed; M7, applicability and limitations adequately discussed.

d No aggregate score or percentage was calculated. Criteria were interpreted at study level according to design, and individual weaknesses were considered during the narrative synthesis.

## Discussion

### Statement of principal findings

This systematic review synthesized 21 heterogeneous studies ­published between 2020 and 2025. Fifteen studies provided direct medical-waste-management evidence, five provided healthcare-sustainability or planetary-health contextual evidence, and one provided transferable waste-system evidence. The synthesis identified five overlapping domains: behavioural compliance and education; workflow and operational optimization; digital and decision-support systems; environmental and economic assessment; and systemic or organizational barriers. Behavioural and educational factors were addressed most frequently, but improvements were not invariably sustained or effective. Modelling and decision-support studies identified potential environmental, economic, and operational benefits, although real-world implementation evidence remained limited. The findings therefore support a multi-level socio-technical interpretation rather than demonstrating the effectiveness of a single Green Hospital intervention model. This pattern is compatible with an HFE-informed socio-technical interpretation, although HFE was applied as an interpretive framework in this review rather than as a consistently evaluated intervention across the included studies [5, 6, 7].

### Strengths and limitations

This review has several strengths, including a comprehensive search strategy, adherence to PRISMA guidelines, and study-level methodological appraisal using criteria appropriate to the included empirical and modelling designs. Nonetheless, limitations include the heterogeneity of study designs, variability in outcome metrics, reliance on self-reported data, and underrepresentation of some geographical regions. The high proportion of reports that could not be retrieved may have reduced the completeness of the evidence base and introduced selection bias. Because the reasons for non-retrieval were not further categorized, the direction and magnitude of this potential bias could not be assessed. Methodological concerns varied by design and included incomplete follow-up or intervention adherence in some trials, limited control of confounding in non-randomized interventions, representativeness and measurement limitations in some single-centre surveys, and inconsistent external validation or sensitivity analysis in modelling studies. In addition, five studies provided contextual healthcare-sustainability or planetary-health evidence and one provided transferable municipal waste-system evidence rather than direct medical-waste-management outcomes. These studies were explicitly identified in Table 1 and were used to inform the wider Green Hospital interpretation, not to estimate direct intervention effectiveness. The overlapping synthesis themes also mean that study counts across categories are not mutually exclusive.

### Interpretation within the context of the wider literature

#### Behavioural and organizational drivers of sustainability

Behavioural compliance was a central determinant of medical-waste-management performance in the direct evidence base. Cross-sectional and observational studies identified deficiencies in knowledge, segregation, institutional policy awareness, infrastructure, and continuing education [9, 23, 25, 29, 31]. Educational interventions improved biomedical-waste knowledge and practices [27, 30], but their effects were not uniformly sustained, and passive educational provision did not necessarily change disposal behaviour [24]. Contextual climate-education and lifestyle studies demonstrated that digital or video-supported interventions could improve environmental knowledge, self-efficacy, or behaviour, but these findings should not be interpreted as direct evidence of improved medical-waste management [22, 28, 36]. Recent studies emphasize the importance of behaviour identification methods, which systematically prioritize the most impactful behaviours and design targeted interventions to achieve meaningful change [10].

Leadership and governance also played a crucial role in enabling sustained compliance. This is consistent with broader evidence from circular economy research in healthcare, which highlights that institutional adoption of sustainable practices depends not only on frontline staff engagement but also on strategic leadership and organizational readiness [11, 16].

#### Technological enablement and innovation

Digital and analytical tools supported several components of sustainable waste management, but the evidence was heterogeneous. This direction is consistent with wider literature describing digitalisation, automated segregation, and data-driven technologies as emerging components of healthcare waste management [8, 12]. IoT-based monitoring and route optimization improved operational indicators in a transferable municipal-waste pilot [20]. Within healthcare-related applications, integrated ERA–MFA assessment supported waste tracking and hotspot identification [3], while Bayesian networks, fuzzy-DEMATEL, and CRITIC–TOPSIS models supported risk analysis, prioritization, and technology selection [4, 21, 33]. These studies demonstrate the potential value of digital monitoring and decision-support methods, but they do not establish that AI-enabled hospital waste systems consistently improve segregation or compliance in routine practice. Technology should therefore be considered an enabling component that requires appropriate data quality, workflow integration, organizational ownership, and user engagement.

#### Environmental and economic integration

Ten studies contributed to the environmental or economic assessment, modelling, circularity, or resource-recovery theme [3, 4, 17, 20, 21, 25, 26, 32–34]. Direct medical-waste studies quantified waste generation and misclassification [18, 25], modelled hazardous-waste quantities and management costs [17], evaluated logistics-related energy costs and transport risks [14, 26], and assessed environmental costs across public hospitals [34]. Other studies examined circular-economy adoption factors, healthcare supply-chain resilience, treatment-technology selection, and integrated environmental-risk and material-flow assessment [3, 4, 13, 21, 33]. Addas *et al.* [20] provided transferable operational evidence from an urban IoT waste system, whereas Rohr *et al.* [32] provided broader planetary-health and circular-resource evidence rather than healthcare-waste outcomes. Overall, the evidence identified both observed and modelled opportunities for reducing environmental burden, improving resource use, and controlling costs; however, benefits were not demonstrated uniformly through real-world hospital implementation studies.

#### Systemic and structural constraints

Seven studies contributed to the systemic and organizational-barriers theme [4, 9, 21, 23, 29, 31, 35]. Frequently reported barriers included weak regulatory enforcement, inadequate financial support, insufficient infrastructure and ­equipment, limited staff training, fragmented organizational responsibility, and poor access to designated storage or treatment resources [19]. Studies from Somalia, rural China, South Africa, India, and Bangladesh demonstrated that governance, funding, stakeholder participation, and institutional capacity influenced segregation, disposal, policy compliance, and adoption of circular-economy practices [4, 9, 23, 29, 31, 35]. Healthcare supply-chain modelling further indicated that sustainability and resilience were sensitive to sourcing, logistics, quality, and dynamic risk-management capacity [21].

### Implications for policy, practice, and research

Hospitals and health systems should prioritize integrated strategies that combine behavioural reinforcement, technological enablement, and governance structures. Continuous professional development, real-time feedback mechanisms, and organizational reinforcement may be more sustainable than isolated training programmes. Investments in digital systems should include interoperability standards and user-centred design to ensure alignment with clinical workflows.

Policymakers should embed circular economy principles into healthcare regulations, incentivize sustainable technology adoption, and provide funding for infrastructure upgrades. Cross-sector collaboration, including partnerships with technology providers, waste management services, and academic institutions, can accelerate knowledge exchange and diffusion of best practices [15].

Future research should focus on longitudinal evaluations of behaviour-change interventions, real-world assessments of IoT and AI-enabled waste systems, and comparative studies across different healthcare contexts. Comprehensive lifecycle assessments are needed to quantify long-term environmental and economic impacts, while modelling studies could guide resource allocation and identify optimal sustainability strategies. These findings are also directly relevant to healthcare quality improvement: inconsistent segregation and disposal practices represent a workflow-reliability and patient-safety concern, given the infection, cytotoxic, and chemical-exposure risks associated with mismanaged medical waste, while digital and decision-support tools that strengthen monitoring and compliance auditing can be understood as quality-assurance mechanisms supporting safer, more reliable waste-handling processes and overall service performance.

## Conclusions

Sustainable medical waste management within the Green Hospital framework requires an integrated socio-technical strategy that combines accurate segregation, continuing behavioural reinforcement, operational optimization, appropriate decision-support technologies, environmental and economic assessment, and enabling governance. The evidence indicates that training can improve knowledge and practice, but its effects may weaken without reinforcement, feedback, infrastructure, and organizational accountability. Modelling and digital approaches offer potential operational and environmental benefits, although real-world implementation evidence remains limited. Systemic barriers relating to regulation, finance, infrastructure, and institutional capacity continue to constrain progress, particularly in resource-limited settings. These findings are compatible with an HFE-informed interpretation of medical-waste systems, but they do not establish the effectiveness of a formal HFE intervention. Future research should prioritize longitudinal, healthcare-specific implementation studies using comparable waste, environmental, economic, and behavioural outcomes.

## Supplementary Material

mzag103_Supplementary_Data

## Data Availability

All data analysed in this study were derived from published, publicly available sources. No new data were generated. Extracted data and synthesis tables are included within the article and its supplementary material.
