# Supplementary material for: Medical waste management within the Green Hospital approach: a systematic review of direct, contextual, and transferable evidence
Source: Int J Qual Health Care. 2026 Aug 3;38(3):mzag103. doi: 10.1093/intqhc/mzag103 (PMC13492086; doi:10.1093/intqhc/mzag103)
Supplement: Supplementary file 1 [file mzag103_supplementary_data.zip › Supplementary File 1/20250827-SR_Protocol_Databases-Turkey.docx]

I. **PRISMA/Protocol**

Inclusion in PRISMA-SR

| **Database** | **Search Terms** | **Notes / Adaptation** |
| --- | --- | --- |
| **PubMed** | (("Waste Management"[Mesh] OR "Medical Waste"[Mesh] OR "Hazardous Waste"[Mesh] OR "Infectious Waste"[Mesh] OR "Biomedical Waste"[All Fields] OR "medical waste"[All Fields] OR "healthcare waste"[All Fields] OR "clinical waste"[All Fields] OR "biohazardous waste"[All Fields] OR "sharps waste"[All Fields] OR "pharmaceutical waste"[All Fields] OR "healthcare waste disposal"[All Fields]) AND ("Hospital Design"[Mesh] OR "Sustainability"[Mesh] OR "Energy Efficiency"[Mesh] OR "Environmental Health"[Mesh] OR "Green Hospitals"[All Fields] OR "green hospital"[All Fields] OR "sustainable healthcare"[All Fields] OR "eco-friendly hospitals"[All Fields] OR "hospital sustainability"[All Fields] OR "environmental hospital practices"[All Fields] OR "green healthcare"[All Fields] OR "waste reduction hospitals"[All Fields] OR "environmental sustainability healthcare"[All Fields]) AND ("waste reduction strategies"[All Fields] OR "waste minimization"[All Fields] OR "hospital recycling programs"[All Fields] OR "sustainable medical waste disposal"[All Fields] OR "eco-hospitals"[All Fields] OR "environmental"[All Fields])) | Full strategy with MeSH and free-text; adapted to PubMed indexing. |
| **Scopus** | TITLE-ABS-KEY("biomedical waste" OR "medical waste" OR "healthcare waste" OR "clinical waste" OR "biohazardous waste" OR "sharps waste" OR "pharmaceutical waste" OR "healthcare waste disposal") AND TITLE-ABS-KEY("green hospital*" OR "sustainable healthcare" OR "eco-friendly hospital*" OR "hospital sustainability" OR "environmental hospital practices" OR "green healthcare" OR "waste reduction in hospitals" OR "environmental sustainability in healthcare") AND TITLE-ABS-KEY("waste reduction strategies" OR "waste minimization" OR "hospital recycling program*" OR "sustainable medical waste disposal" OR "eco-hospital*" OR "environmental") | Uses TITLE-ABS-KEY fields; truncation (*) captures variations. |
| **Web of Science** | TS=("biomedical waste" OR "medical waste" OR "healthcare waste" OR "clinical waste" OR "biohazardous waste" OR "sharps waste" OR "pharmaceutical waste" OR "healthcare waste disposal") AND TS=("green hospital*" OR "sustainable healthcare" OR "eco-friendly hospital*" OR "hospital sustainability" OR "environmental hospital practices" OR "green healthcare" OR "waste reduction in hospitals" OR "environmental sustainability in healthcare") AND TS=("waste reduction strategies" OR "waste minimization" OR "hospital recycling program*" OR "sustainable medical waste disposal" OR "eco-hospital*" OR "environmental") | TS=Topic (title, abstract, keywords); similar to Scopus. |
| **Cochrane Library** | ([biomedical waste:ti,ab,kw OR medical waste:ti,ab,kw OR healthcare waste:ti,ab,kw OR clinical waste:ti,ab,kw OR biohazardous waste:ti,ab,kw OR sharps waste:ti,ab,kw OR pharmaceutical waste:ti,ab,kw OR healthcare waste disposal:ti,ab,kw]) AND ([green hospital*:ti,ab,kw OR sustainable healthcare:ti,ab,kw OR eco-friendly hospital*:ti,ab,kw OR hospital sustainability:ti,ab,kw OR environmental hospital practices:ti,ab,kw OR green healthcare:ti,ab,kw OR waste reduction hospitals:ti,ab,kw OR environmental sustainability in healthcare:ti,ab,kw]) AND ([waste reduction strategies:ti,ab,kw OR waste minimization:ti,ab,kw OR hospital recycling program*:ti,ab,kw OR sustainable medical waste disposal:ti,ab,kw OR eco-hospital*:ti,ab,kw OR environmental:ti,ab,kw]) | Uses :ti,ab,kw for titles, abstracts, and keywords. |
| **OpenGrey** | ("medical waste" OR "healthcare waste" OR "biomedical waste") AND ("green hospital" OR "sustainable healthcare" OR "eco-hospital") AND ("waste reduction" OR "hospital recycling" OR "waste minimization") | Simplified strategy due to limited operators. |

| **Database** | **Search String** | **Date Searched** | **TN** |
| --- | --- | --- | --- |
| **PubMed** | ("Waste Management"[MeSH Terms] OR "Medical Waste"[MeSH Terms] OR "Hazardous Waste"[MeSH Terms] OR "Biomedical Waste"[All Fields] OR "Medical Waste"[All Fields] OR "healthcare waste"[All Fields] OR "clinical waste"[All Fields] OR "biohazardous waste"[All Fields] OR "sharps waste"[All Fields] OR "pharmaceutical waste"[All Fields] OR "healthcare waste disposal"[All Fields]) AND ("Environmental Health"[MeSH Terms] OR "Green Hospitals"[All Fields] OR "green hospital"[All Fields] OR "sustainable healthcare"[All Fields] OR "eco-friendly hospitals"[All Fields] OR "hospital sustainability"[All Fields] OR "environmental hospital practices"[All Fields] OR "green healthcare"[All Fields] OR "waste reduction hospitals"[All Fields] OR "environmental sustainability healthcare"[All Fields]) AND ("waste reduction strategies"[All Fields] OR "waste minimization"[All Fields] OR "hospital recycling programs"[All Fields] OR "sustainable medical waste disposal"[All Fields] OR "eco-hospitals"[All Fields] OR "environmental"[All Fields]) AND (y_5[Filter]) AND (published_article[Filter] OR author_manuscript[Filter])    **Note:** The PubMed interface displayed 420 results at the time of the search. However, the citation file exported from PubMed and imported into EndNote contained 409 records. Therefore, 409, the number of records that actually entered the screening process, is reported in the PRISMA flow diagram. | 20250827  @11:30 | 420 displayed; 409 exported/ imported |
| **Scopus** | TITLE-ABS-KEY("biomedical waste" OR "medical waste" OR "healthcare waste" OR "clinical waste" OR "biohazardous waste" OR "sharps waste" OR "pharmaceutical waste" OR "healthcare waste disposal") AND TITLE-ABS-KEY("green hospital*" OR "sustainable healthcare" OR "eco-friendly hospital*" OR "hospital sustainability" OR "environmental hospital practices" OR "green healthcare" OR "waste reduction in hospitals" OR "environmental sustainability in healthcare") AND TITLE-ABS-KEY("waste reduction strategies" OR "waste minimization" OR "hospital recycling program*" OR "sustainable medical waste disposal" OR "eco-hospital*" OR "environmental")   | 20250827  @11:42 | 28 |
| **WOS** | ("biomedical waste" OR "medical waste" OR "healthcare waste" OR "clinical waste" OR "biohazardous waste" OR "sharps waste" OR "pharmaceutical waste" OR "healthcare waste disposal") AND ("green hospital*" OR "sustainable healthcare" OR "eco-friendly hospital*" OR "hospital sustainability" OR "environmental hospital practices" OR "green healthcare" OR "waste reduction in hospitals" OR "environmental sustainability in healthcare") AND ("waste reduction strategies" OR "waste minimization" OR "hospital recycling program*" OR "sustainable medical waste disposal" OR "eco-hospital*" OR "environmental")   | 20250827  @12:05 | 32 |
| **Cochrane Library** | biomedical waste OR medical waste OR healthcare waste OR clinical waste OR biohazardous waste OR sharps waste OR pharmaceutical waste OR healthcare waste disposal AND green hospital* OR sustainable healthcare OR eco-friendly hospital* OR hospital sustainability OR environmental hospital practices OR green healthcare OR waste reduction hospitals OR environmental sustainability in healthcare AND waste reduction strategies OR waste minimization OR hospital recycling program* OR sustainable medical waste disposal OR eco-hospital* OR environmental     | 20250827  @11:50 | 10036 |
| **CINAHL**  **(EBSCOhost)** | ("biomedical waste" OR "medical waste" OR "healthcare waste" OR "clinical waste" OR "biohazardous waste" OR "sharps waste" OR "pharmaceutical waste" OR "healthcare waste disposal") AND ("green hospital*" OR "sustainable healthcare" OR "eco-friendly hospital*" OR "hospital sustainability" OR "environmental hospital practices" OR "green healthcare" OR "waste reduction in hospitals" OR "environmental sustainability in healthcare") AND ("waste reduction strategies" OR "waste minimization" OR "hospital recycling program*" OR "sustainable medical waste disposal" OR "eco-hospital*" OR "environmental")   | 20250827  @12:11 | 12 |
| **EMBASE** | ("biomedical waste" OR "medical waste" OR "healthcare waste" OR "clinical waste" OR "biohazardous waste" OR "sharps waste" OR "pharmaceutical waste" OR "healthcare waste disposal") AND ("green hospital*" OR "sustainable healthcare" OR "eco-friendly hospital*" OR "hospital sustainability" OR "environmental hospital practices" OR "green healthcare" OR "waste reduction in hospitals" OR "environmental sustainability in healthcare") AND ("waste reduction strategies" OR "waste minimization" OR "hospital recycling program*" OR "sustainable medical waste disposal" OR "eco-hospital*" OR "environmental")   | 20250827  @12:25 | 45 |
