# Supplementary material for: Medical waste management within the Green Hospital approach: a systematic review of direct, contextual, and transferable evidence
Source: Int J Qual Health Care. 2026 Aug 3;38(3):mzag103. doi: 10.1093/intqhc/mzag103 (PMC13492086; doi:10.1093/intqhc/mzag103)
Supplement: Supplementary file 1 [file mzag103_supplementary_data.zip › Supplementary File 1/Table S1.docx]

| Table S1. Search strategy for PubMed | | |
| --- | --- | --- |
| Focused question | | **What direct, contextual, and transferable evidence informs medical waste management within the Green Hospital framework?** |
| Search strategy | (#1)  Medical Waste Management | ("Medical Waste"[Mesh] OR "Biomedical Waste"[Mesh] OR "Hazardous Waste"[Mesh] OR "Infectious Waste"[Mesh] OR medical waste OR healthcare waste OR clinical waste OR biohazardous waste OR sharps waste OR pharmaceutical waste OR healthcare waste disposal OR "Waste Management"[Mesh]) |
|  | (#2)  Green Hospital Approach | ("Green Hospital" OR "Sustainable Hospital" OR "Eco-Hospital" OR "Hospital Design"[Mesh] OR "Sustainability"[Mesh] OR "Energy Efficiency"[Mesh] OR "Environmental Health"[Mesh] OR sustainable healthcare OR green healthcare OR environmental hospital practices OR environmental sustainability in healthcare OR waste reduction in hospitals) |
|  | (#3)  Integration of Medical Waste Management in Green Hospitals | (waste minimization OR waste reduction strategies OR hospital recycling programs OR recycling OR sustainable medical waste disposal OR environmental policies in healthcare OR eco-hospitals) |
|  | (#4)  Combined Search 1 | #1 AND #2 |
|  | (#5)  Combined Search 2 | #1 AND #3 |
|  | (#6)  Combined Search 3 | #2 AND #3 |
|  | (#7)  Combined Search 4 | #1 AND #2 AND #3 |
|  | Example Search String: | ("Medical Waste" OR "Biomedical Waste" OR "Healthcare Waste") AND ("Green Hospital" OR "Sustainable Hospital" OR "Eco-Hospital") AND ("Waste Management" OR "Waste Minimization" OR "Recycling" OR "Disposal") |
